# Pneumopericardium after pericardiocentesis for malignant pericardial effusion

**DOI:** 10.1093/ehjcr/ytag713

**Published:** 2026-09-23

**Authors:** Machiko Miyoshi, Kanae Hasegawa, Kentaro Ishida, Hiroshi Tada

**Affiliations:** Department of Cardiovascular Medicine, Faculty of Medical Sciences, University of Fukui, 23-3, Matsuokashimoaizuki, Eiheiji-cho, Yoshida-gun, Fukui 910-1193, Japan; Department of Cardiovascular Medicine, Faculty of Medical Sciences, University of Fukui, 23-3, Matsuokashimoaizuki, Eiheiji-cho, Yoshida-gun, Fukui 910-1193, Japan; Department of Cardiovascular Medicine, Faculty of Medical Sciences, University of Fukui, 23-3, Matsuokashimoaizuki, Eiheiji-cho, Yoshida-gun, Fukui 910-1193, Japan; Department of Advanced Cardiovascular Therapeutics, Faculty of Medical Sciences, University of Fukui, Fukui, Japan

**Keywords:** Complication, Pericardiocentesis, Pericardial effusion, Pneumopericardium

## Summary

Pneumopericardium after pericardiocentesis is a rare but recognized complication. In this case, computed tomography clarified the true intrapericardial position of the drainage catheter and confirmed the diagnosis when chest radiography created the misleading impression of an intracardiac catheter.

## Case description

A 49-year-old woman with recurrent malignant pericardial effusion from metastatic renal cell carcinoma underwent repeat pericardiocentesis for progressive dyspnoea. Chest X-ray showed cardiomegaly (*Figure 1A*). Transthoracic echocardiography (*Figure 1B–C*)) before the procedure showed a large pericardial effusion with a swinging heart and diastolic collapse.

After echocardiographic confirmation of a safe site, pericardiocentesis was performed via the subxiphoid approach under echocardiographic guidance. After the needle entered the pericardial space, position was confirmed by fluid aspiration. A guidewire was advanced, and fluoroscopy confirmed its intrapericardial position. A bubble test was not performed. The catheter was then advanced over the guidewire, and 400 mL of yellow-green fluid was drained. Chest X-ray obtained 30 min later showed marked reduction in the cardiothoracic ratio (*Figure 1D*), whereas the pericardial contour remained unchanged, making the indwelling catheter appear to lie within the cardiac silhouette. Because this raised concern of an intracardiac catheter, echocardiography was repeated but poorly visualized the heart, likely because intrapericardial air interfered with imaging. Therefore, computed tomography (*Figure 1E–G*) was performed to clarify the catheter position, demonstrating that the catheter lay beneath the heart within the pericardial space rather than intracardially, with the drained fluid replaced by air and an air–fluid level, confirming pneumopericardium.^1^

She remained haemodynamically stable. Continuous suction at 12 cmH_2_O was started. As chest X-ray the following day showed improvement, suction was reduced to 10 cmH_2_O and tapered. As only slight pneumopericardium remained on the X-ray (*Figure 1H*), the catheter was removed on Day 5. Serial imaging showed progressive resolution of the pneumopericardium and effusion, with no worsening on chest radiography (*Figure 1I*) and echocardiography (*Figure 1J-K*) at 30 days after drain removal.

**Figure 1:**
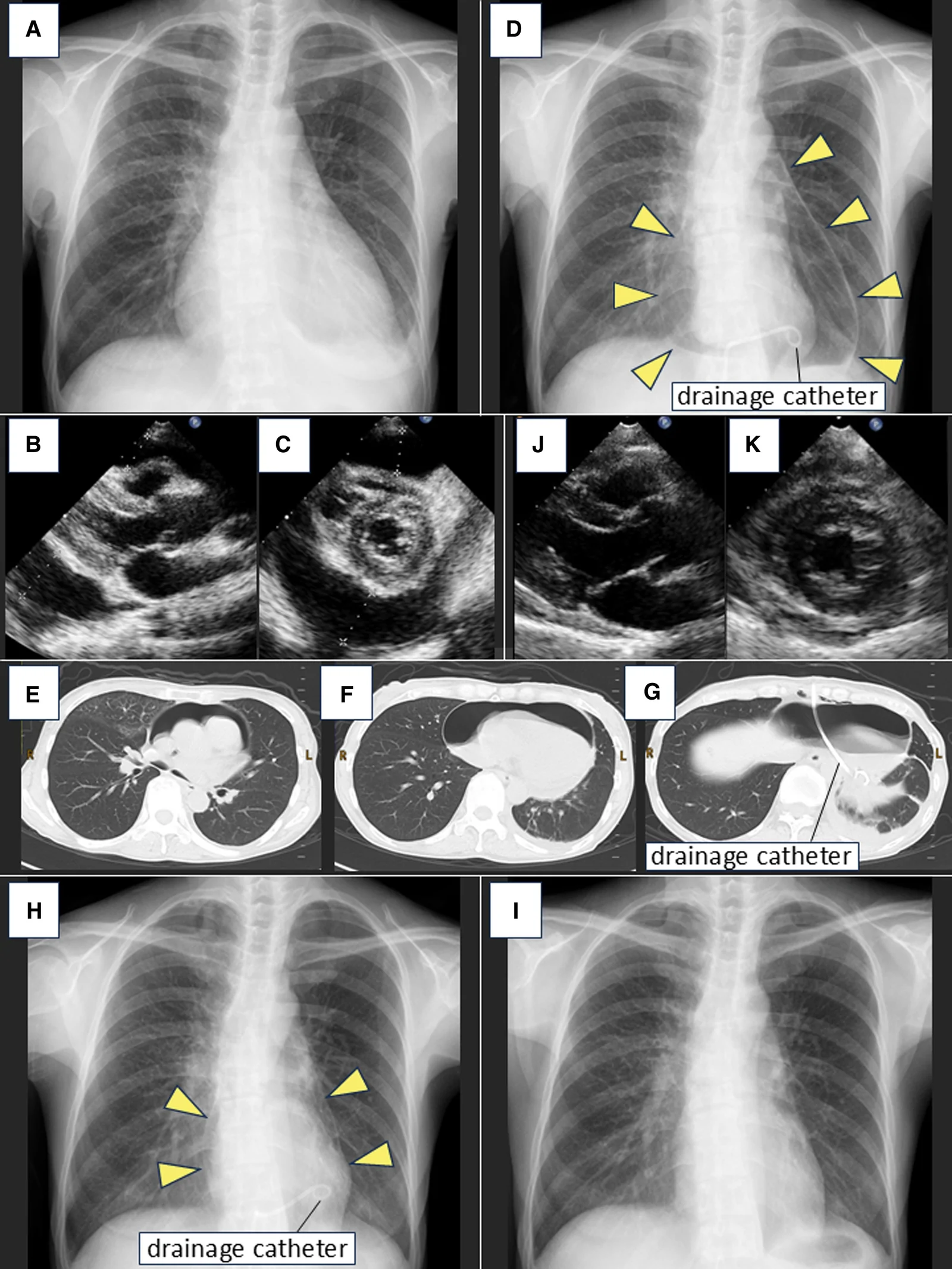
Pneumopericardium after pericardiocentesis. (*A*) Chest radiograph before drainage showing marked cardiomegaly. (*B*, *C*) Transthoracic echocardiography before the procedure showing a large pericardial effusion with a swinging heart and diastolic chamber collapse. (*D*) Chest radiograph 30 min after drainage showing a reduced cardiothoracic ratio with intrapericardial air (arrowheads); the drainage catheter appears to lie within the cardiac silhouette. (*E–G*) Axial computed tomography demonstrating an air–fluid level within the pericardial space and the drainage catheter located beneath the heart within the pericardial space rather than intracardially. (*H*) Chest radiograph showing only slight residual pneumopericardium (arrowheads) before catheter removal on Day 5. (*I*) Chest radiograph and (*J*, *K*) transthoracic echocardiography at 30 days after catheter removal, showing resolution of the pneumopericardium and pericardial effusion without recurrence.

## Data Availability

The data underlying this article are available within the article. Further anonymized data are available from the corresponding author on reasonable request.

## Notes

**Consent:** Written informed consent for publication of this case report and all accompanying images was obtained from the patient’s next of kin, in accordance with COPE guidelines.
